# Integrating specialist ophthalmic services into emergency medical teams

**DOI:** 10.2471/BLT.20.255786

**Published:** 2020-09-03

**Authors:** David McMaster, Gerry Clare

**Affiliations:** a University of Nottingham School of Medicine, Queen’s Medical Centre, Nottingham, NG7 2UH, England.; b Moorfields Eye Hospital NHS Foundation Trust, London, England.

Ocular injuries and disruption to ophthalmic health-care services are common features of sudden-onset disasters, and often result in life-changing consequences for those affected. Each year, hundreds of millions of people are affected by large-scale disruptions, such as earthquakes or conflicts,^1^ that cause major human and material losses and overwhelm the ability of communities to cope with health-care demands. In response, national and international emergency medical teams consisting of various health professionals deploy to disaster zones to minimize loss of life, prevent disability and restore stability.

The World Health Organization (WHO) classifies emergency medical teams into three types. Type 1 provides outpatient services, and types 2 and 3, respectively, provide acute and complex inpatient care and have the capacity to accommodate additional specialized teams as required by the host country and the scope of the medical teams’ mission (Fig. 1).^2^ As a result of the time it takes for foreign emergency medical teams to arrive in-country and respond to disasters, opportunities to provide acute trauma care can be missed or delayed.^3^^,^^4^

**Fig. 1:**
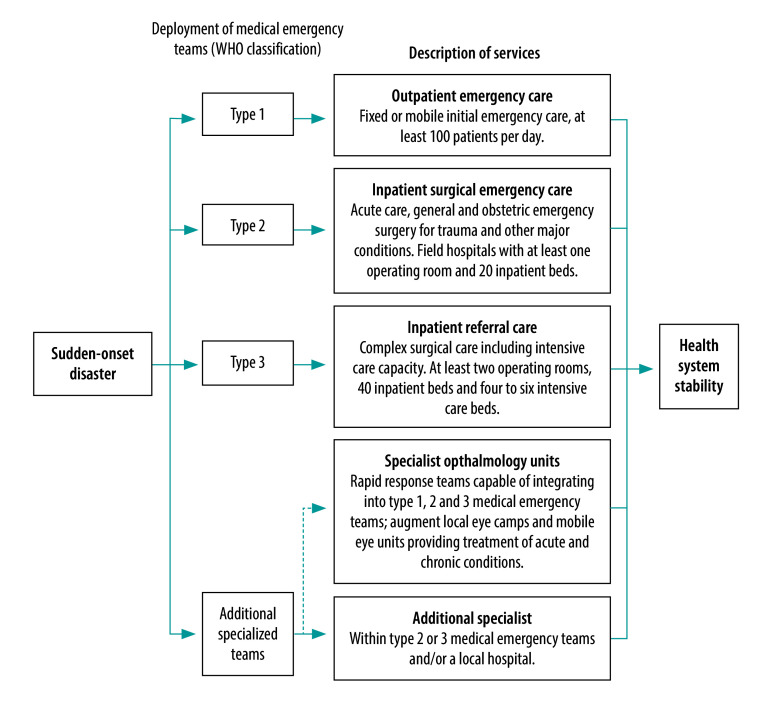
Integrating specialist ophthalmic services into WHO-classified emergency medical teams

WHO: World Health Organization.

During such events, qualified health-care workers trained in the management of emergency eye conditions, including ocular trauma, normally provide ophthalmic care within pre-existing structures, with minimal added capacity. However, ocular conditions that non-specialists cannot deal with inevitably arise. In view of the potential for increased number of eye-care cases, severity of ocular injuries, and the breakdown of normal health-care provision, we advocate increasing specialist ophthalmic services within emergency medical teams.

## Eye-care demands

The role of emergency medical teams is to provide and maintain essential health care, including managing the direct consequences of the crisis, such as mass injuries and subsequent disease outbreaks, coincidental medical emergencies and pre-existing chronic health conditions. Conceivably, ophthalmic surgeons and allied health-care workers could contribute to the emergency response across all of these domains.

The demand for ophthalmic care varies with the nature of the challenge. In armed conflict, injuries to the eye occur disproportionately to its surface area, and in recent conflicts the incidence of combat-related ocular injuries has continued to increase.^5^ Studies estimate that 13% of military casualties in modern wars include ocular injuries.^6^ Among civilian casualties, children are particularly vulnerable to blast injuries, with 80% experiencing penetrating injuries to the head, compared to only 31% of adult victims.^7^ The deployment of explosive devices in densely populated areas means that ophthalmic surgeons who can treat often complex ocular injuries are needed. Novel weapons such as blinding lasers may further complicate the overall situation. 

Damage to health infrastructure severely affects health service delivery and can set back the improvement of a health system by years. For example, the Great East Japan Earthquake of 2011 triggered powerful tsunami waves and caused almost 20 000 casualties, widespread evacuation and complete or partial destruction of nearly 400 000 buildings along the coastline, including many hospitals.^8^ A mobile eye unit boosted relief efforts by temporarily replacing local services, reaching 2070 people in 39 days, performing ophthalmic examinations and distributing medical supplies including eye drops and contact lenses.^9^ The major ophthalmic needs included pre-existing chronic eye conditions such as cataract and glaucoma, with approximately less than 1% of patients requiring treatment for emergencies such as ocular trauma and corneal abrasions.^9^ In contrast, acute ocular injuries can be the main concern in certain disasters, such as the Bhopal (India) gas tragedy of 1984 in which the accidental release of methyl isocyanate into the atmosphere caused widespread chemical eye injuries.

Ocular complications may precipitate an urgent need for ophthalmic care during outbreaks of contagious disease, which can vastly overwhelm community resources. During the 2013–2016 Ebola virus disease outbreak in West Africa, survivors developed often avoidable sight loss as a result of cataract, glaucoma and retinal detachment caused by intraocular inflammation. This outcome posed a new challenge for nongovernmental organizations, which were unprepared for this eventuality. Elsewhere, the measles outbreak in the Pacific region in 2019 triggered mass prophylactic treatment with vitamin A to prevent unnecessary sight loss from secondary ophthalmic complications.

## Integrating services

Local reconnaissance of disaster areas to estimate service gaps and injury types and numbers is essential to planning effective service delivery. Distinct patterns of injury can dominate depending on the nature of the incident, which may require a tailored response. For example, following the Nepal Earthquake of 2015, significant numbers of traumatic ocular injury were recorded, indicating a need for ophthalmic surgical teams.^10^

Effective eye trauma care is often time critical, and military units such as the United States of America Air Force Mobile Ophthalmic Surgery Team have the capacity to mount a response to emergencies at short notice.^11^ Deploying with backpacks containing portable equipment including a small operating microscope, the team aims to mobilize within two hours of notification, and can perform approximately 10 major eye operations and 100 outpatient consultations. The team can travel by commercial airlines with checked luggage and support relief efforts while a more focused response can be coordinated and set up in-country.

Providing ophthalmologists and ophthalmic personnel is essential to manage a variety of ophthalmic conditions that might become sight threatening if not addressed promptly. Inclusion of these specialists within emergency medical team responses allows the possibility of best outcomes for ophthalmic patients and reduces the workload of other emergency medicine physicians, general surgeons and allied professionals, allowing them to concentrate on their own skill sets.

As the emergency relief phase settles, the team’s presence can be invaluable to prepare and contribute to the recovery phase of the affected country’s health system. Emergency medical teams that have invested in local infrastructure (for example type 2 field hospitals) are in a good position to contribute to the transition back to local services by supporting the management of chronic conditions (for instance cataracts and glaucoma) and mitigating the incidental increase in sight loss that might occur as a result of the disrupted service delivery.

In some disasters, liaising with local health-care structures to coordinate an effective ophthalmic response might be possible. Eye camps, for example, are usually temporary screening and treatment centres that provide an effective platform for combating blindness in resource-poor settings. These centres could be strengthened with staff and equipment to form type 2 emergency medical team facilities in the event of a disaster. In addition, mobile eye units – ranging from well-equipped four-wheel drive utility vehicles to large specialist lorries with onboard operating theatres – might be deployed to locations cut off by natural disasters to offer essential ophthalmic services.

Recent advances make it possible to use mobile phone applications to accurately record visual acuity and capture images of the retina. In addition, measurement of intraocular pressure can now be achieved easily using portable equipment. These advances, and the increased use of telemedicine, are revolutionizing eye care in resource-poor areas and could be harnessed by health-care workers to provide detailed information on ophthalmic conditions to centralized ophthalmic teams as part of the response to a disaster.

## Conclusions

We believe that awareness of the importance of eye care should inform the response to all sudden-onset disasters. To ensure basic eye care can be offered, we recommend a minimum proportion of emergency medical teams personnel to be trained to diagnose and manage several acute eye conditions using essential ophthalmic equipment and consumables. In addition, we recommend the use of specialist ophthalmology units able to deploy independently or attach to emergency medical teams. These specialist cells should consist of small surgical teams for rapid deployment, with the ability to use and boost local eye camps and mobile eye units, complementing current WHO emergency medical team structures (Fig. 1).

Combined with working operating theatres, these measures would help both in the initial management of ocular trauma and in returning normal service delivery to affected countries. Providing skilled ophthalmic care after a disaster may significantly facilitate the development of local eye health resources. Lessons can be learnt from efforts to rebuild the ophthalmic health-care system in post-conflict areas such as Timor-Leste, where international organizations worked with local government and services to optimize resources.^12^

Sudden-onset disasters expose populations to an increased risk of eye injuries and complications that can easily overwhelm local resources. Universal health coverage relies on global efforts to support damaged eye health systems. Integrating specialist ophthalmic services into emergency medical teams may be an effective way of managing ocular injuries, reducing unnecessary sight loss and supporting a transition back to local governance as infrastructure and human resources are rebuilt. Ocular health is an integral part of well-being, and ophthalmic care in the aftermath of a disaster should not be neglected.
